# From legacy to integration in the Global Polio Eradication Initiative: looking back to look forward

**DOI:** 10.1136/bmjgh-2023-014758

**Published:** 2024-05-08

**Authors:** Svea Closser, Abigail H Neel, Sue Gerber, Olakunle Alonge

**Affiliations:** 1 International Health, Johns Hopkins University Bloomberg School of Public Health, Baltimore, Maryland, USA; 2 Independent Consultant, Truchas, New Mexico, USA; 3 Sparkman Center for Global Health, The University of Alabama, Birmingham, Alabama, USA

**Keywords:** Poliomyelitis, Qualitative study, Global Health, Health systems, Immunisation

## Abstract

**Introduction:**

The Global Polio Eradication Initiative (GPEI) is a global single-disease programme with an extensive infrastructure in some of the world’s most underserved areas. It provides a key example of the opportunities and challenges of transition efforts—the process of shifting from donor-funded, single-disease programmes to programmes with more integrated and sustainable programmatic and funding streams. Our goal is to closely analyse the social and political dynamics of the polio transition in the 2010s to provide insights into today, as well as lessons for other programmes.

**Methods:**

We conducted semistructured interviews with GPEI officials involved in transition planning across GPEI partner agencies (n=11). We also drew on document review and interviews with national and subnational actors in Nigeria, India, Ethiopia and the Democratic Republic of the Congo. We inductively analysed this material to capture emergent themes in the evolution of transition activities in the GPEI.

**Results:**

Since the mid-2010s, GPEI actors expressed concern that polio’s assets should not be lost when polio was eradicated. Planning for polio’s legacy, however, proved complicated. The GPEI’s commitment to and focus on eradication had taken precedence over strong collaborations outside the polio programme, making building alliances for transition challenging. There were also complex questions around who should be responsible for the transition process, and which agencies would ultimately pay for and deliver polio-funded functions. Current efforts to achieve ‘integration’ both have great promise and must grapple with these same issues.

**Discussion:**

Within the GPEI, relinquishing control to other programmes and planning for significant, long-term funding for transition will be central to achieving successful integration and eventual transition. Beyond polio, other vertical programmes can benefit from going beyond transition ‘planning’ to integrate transition into the initial design of vertical programmes.

**WHAT IS ALREADY KNOWN ON THIS TOPIC**

- Major transitions from the President’s Emergency Fund for AIDS Relief to Gavi have shared common challenges related to mobilising resources and political will, integrating service delivery, sustaining equity for vulnerable populations and developing institutional capacity.

**WHAT THIS STUDY ADDS**

- It is important for vertical programmes to build robust relationships early with other programmes, particularly government health systems, to build collaborations that can eventually facilitate transition.

**HOW THIS STUDY MIGHT AFFECT RESEARCH, PRACTICE OR POLICY**

- Relinquishing some control over programmes, and providing robust funding streams, will be key to the successful integration of essential health systems functions created by the polio programme.

## Introduction

Transitions of major global public health programmes from international to national funding and control are complex endeavours. Major transitions from PEPFAR (the President’s Emergency Fund for AIDS Relief) to Gavi (the Vaccine Alliance) have shared common challenges related to mobilising resources and political will, integrating service delivery, sustaining equity for vulnerable populations and developing institutional capacity.^1–4^

The Global Polio Eradication Initiative (GPEI), a huge single-disease vertical public health programme that sought to eradicate poliovirus globally by the year 2000, shares many of these same challenges. As an eradication programme, the expectation was always that the GPEI would end along with the end of polio–the idea was that if total eradication of poliovirus was achieved, the end of polio would be inherently sustainable, even if the structures that led to eradication disappeared. This led many to see sustainability as baked into the very structure of eradication, described by some as ‘the ultimate investment in both equity and sustainability—it is for everyone and forever’.^5^ The assumption within the polio programme and among its donors—one that can be clearly seen in its financial projection documents over the years—was that donors’ financial commitments to the GPEI would end along with the end of polio.^6 7^

The eradication goal meant that the efforts of the GPEI were seen as sustainable, not because of the public health systems they might build, but because of the promise that they would end polio permanently. Of course, much has been built by the GPEI. Studies have documented the impressive gains made by the polio programme to develop surveillance and monitoring and evaluation systems, strengthen supply chains and health information systems, and innovate strategies for community outreach.^8–10^ Advocates of the polio programme saw this type of system strengthening as a ‘desirable secondary gain’ of eradication investments^11^ and hoped these functions might find a programmatic home once the job of eradicating polio was finished and funds withdrawn.

But in the long effort to eradicate, the GPEI has become more complexly entwined with national health systems and health service endeavours than it anticipated, having built a complex and far-reaching infrastructure, one with expenditures of hundreds of millions of dollars a year in some countries.^12^ For many years, the programme expended over US$1 billion a year in total, concentrated in areas of the world with the weakest health systems.^13^ This has meant that the drawdown of polio infrastructure may have profound consequences for the sustainability of health outcomes beyond the polio programme, particularly in countries facing simultaneous reductions in external assistance for health.^14^

The GPEI transition is also occurring in the context of larger current debates in global public health. As a global eradication programme, the GPEI was—to a degree that exceeds even most other large single-disease global health programmes—organised in a way where regulations and standards were made in centres of power at the global level (largely Geneva, but also Atlanta, New York and Seattle) and implemented in a largely standardised and centrally controlled way in countries across the world.^15 16^ Beyond the very practical implications of managing a transition, the GPEI’s experiences may resonate with other efforts to move from a global health landscape dominated by Global North institutions into a landscape where a broader variety of actors share in both funding and control of programmes. In particular, it offers an example of the deep ethical and practical complexity of this process when ‘localisation’ is driven by Global North donors and stakeholders.

This paper analyses the experience of the polio transition effort in the 2010s. Currently, the polio programme is again engaging with questions of transition, both through a transition Action Plan and through an increasing focus on integrating its activities with other services.^17–19^ Our goal in this paper is to provide insights into the way forward in the present and lessons for other programmes. In particular, we focus on the social relations and power dynamics of transition efforts. Other papers have helpfully described in detail the technical and funding requirements for polio transition.^20^ Yet the critical dynamics in transition programmes are not just technical and financial—they are also relational and social. Close attention to all of these factors will be critical in the transition of polio and other single-disease vertical programmes.

## Methods

This paper is part of a larger study, Synthesis and Translation of Research and Innovations from Polio Eradication (STRIPE).^21^ The topic of this paper was suggested to us by members of STRIPE’s Technical Advisory Committee (TAC), which includes a range of polio eradication stakeholders. It was clear based on conversations with TAC members that GPEI’s experience with the transition was an important process to document, with many potential lessons for other programmes.

In 2021, we purposively sampled and interviewed global-level respondents centrally involved in transition planning (n=11). Respondents worked for a range of GPEI partner agencies, including WHO (the World Health Organization), UNICEF, CDC (the US Centers for Disease Control and Prevention), Rotary, Gavi and several donor agencies. We purposively sampled respondents who were perceived as having been on ‘opposite sides’ (as one of our respondents put it) of various debates surrounding transition. Our goal was to gain as clear an understanding as possible of the dynamics and constraints that characterised the transition effort at the international level, from multiple perspectives. Our sample aimed not for statistical representation but for insights from those with the deepest familiarity with our study topic.^22 23^

The first author, who knew some but not all respondents from previous work, conducted these interviews, using a semistructured interview guide that explored respondents’ experiences with polio transition planning and implementation. We transcribed the interviews and removed identifying information.

We also drew on national and subnational interviews with polio staff in Nigeria (n=27), Ethiopia (n=30), India (n=25) and the DRC (Democratic Republic of the Congo) (n=23), conducted in 2019. These included government officials and staff as well as staff contracted by partner agencies (most commonly WHO or UNICEF). These interviews were conducted by researchers based in these respective countries. The interviews were not focused on transition but asked about challenges, strategies, and lessons learnt, and a substantial number of respondents discussed transition-related issues in their responses. We also reviewed grey and published literature relevant to GPEI legacy, transition and integration planning.

We analysed the international-level interviews through inductive coding in MAXQDA. We identified emergent themes about the dynamics of implementing transition policies, and the evolution of transition activities over the course of the GPEI from its inception in 1988 to the present. The first author developed the initial codebook through a process of open coding, identifying key themes across the interviews. We also had codes relating to the time period being discussed. The second author reviewed this codebook, discussing the themes with the first author and suggesting revisions and refinements. Both the first and second author then coded the interviews with these themes. We discussed our work on a regular and ongoing basis as we worked, refining our codes and themes and discussing the dynamics at play.

Although we interviewed individuals who were perceived by our respondents to have had opposing viewpoints in the debates surrounding transition, we were particularly struck by how consistent the narrative was across most of our interviews. In places where there were significant differences of opinion on a particular topic or theme, we have noted this in the text.

The national and subnational-level interviews had previously been coded in the qualitative analysis programme Dedoose; the previous coding that had been done included a code for material related to transition. For each of the four countries, a different analyst conducted a close read of the material coded as relating to transition, and authored an extensive memo describing transition dynamics within each study country.^24^ We engaged in weekly review sessions to discuss findings across countries.

We draw primarily on interviews with global-level respondents in the body of this paper, and national and subnational-level respondents in the country-specific boxes.

The authors of this study have held a variety of roles in the polio eradication effort; the first author has been by turn an observer and a participant in polio eradication activities for nearly 20 years. As we describe below, many of our respondents in this paper were very self-critical about what was to them, in retrospect, a critical missed opportunity of vision, planning and relationship building for transition early in the programme. The authors of this paper have no moral or intellectual high ground here. Our previous work has not considered transition in any significant depth, and we did not identify it as a centrally important research topic until prompted to do so by our TAC. We share with our respondents in this paper a feeling that we did not understand the complexity of the dynamics at play, or the importance of building foundations for transition, in the earlier years of the programme. Much of the impetus, for us, behind the work in this paper is to provide these insights to others in other programmes.

### Patient and public involvement

As this study focused on understanding the internal dynamics of policy-making, patients and the public were not involved in the research process.

### ‘We cannot wake up tomorrow and have these resources lost’

When polio eradication was adopted by the World Health Assembly as a goal in 1988, the plan was to eradicate polio with a lean and ephemeral infrastructure. This is in fact what happened in many areas of the world that at the time had stronger health systems: the Americas eliminated polio in 1994 using supplemental immunisation campaigns held at temporary community vaccination posts staffed by Rotarians and other volunteers.^25^ The idea in the Americas was that polio would be a ‘banner disease’ that would galvanise public support for health systems more broadly.^26^ This proved largely to be the case. When the minimal polio eradication infrastructure in the Americas evaporated in the 1990s, it left largely beneficial impacts on health systems in its wake.^27^

Yet in sub-Saharan Africa and South Asia, polio proved much harder to eliminate, and over time, GPEI’s investments in infrastructure and staffing in persistently polio-endemic countries expanded. The GPEI built a world-class global surveillance system for polio, with labs supported by the CDC and extensive human resources staffed by WHO. The operating costs of this high-quality global surveillance system are well over US$100 million per year.^12^ In addition, the GPEI built extensive social mobilisation networks for polio eradication in many countries, funded and staffed by UNICEF.

These networks were strongest in areas where national health systems were the weakest.^8 28^ In many settings, these resources were also being used to facilitate health services beyond polio.^8 29 30^

In the mid-2010s, polio eradication planners in the global hubs of Geneva, Atlanta and New York began to think ahead, with some concern, about what would happen to the polio programme’s infrastructure once polio was eradicated. Our respondents said they had been concerned about the future of these extensive and effective systems. One reflected:

I think the biggest fear was just that a switch would be flipped… Those learnings, that intellectual capital, that experience, those tools, those people that had invested so much would just be lost… The amount of resources and insight in those communities, it’s not just people, it’s social maps, it’s micro plans… in some of the most underserved places in the world.

The dissolution of the Smallpox Eradication Programme was a cautionary tale for our respondents.^28^ They noted that the deep contextual epidemiological and social knowledge and capacity that had enabled the eradication of smallpox was lost to national public health structures once smallpox was eradicated. This was, our respondents commented, because the Smallpox Eradication Programme had not planned for the continued funding or transition of those resources. One said, ‘The whole thing collapsed—and that should not happen to polio.’

There was true zeal among many of our respondents that the polio programme’s resources and knowledge be brought to bear on broader health goals. One commented:

At the global level, I felt a real pressure for this to work, like it *must* happen. We cannot wake up tomorrow and have these resources lost…

An unsuccessful transition in just one country will probably mean people will die.… A transition *has to* succeed in every country.

A concern that failure to transition polio resources in the world’s most fragile states could lead to the wholesale collapse of their immunisation systems was broadly shared by many of our respondents as well as members of the Polio Eradication Independent Monitoring Board (IMB). The IMB used the word ‘legacy’ to describe the need to ensure that the resources the polio programme had created were sustained posteradication.

The GPEI’s 2013–2018 strategic plan included a commitment ‘to ensure that the investments made to eradicate poliomyelitis contribute to future health goals, through a programme of work to systematically document and transition the knowledge, lessons learnt and assets of the GPEI’.^31^ To ensure this, the Polio Legacy Management Group, which included representatives from across the global polio partnership, was created to ‘maintain’, ‘mainstream’ and ‘transition’ polio ‘capacities, processes and assets’.^28^

### Command, control, eradicate

Support for Routine Immunisation (RI) programmes has, on paper, been at the core of the GPEI’s mission since its inception. Yet attention to strengthening RI was sometimes peripheral in practice, for several reasons. GPEI leadership—particularly in the early 2000s when polio supplemental immunisation campaigns ramped up globally—felt that increasing RI coverage was the responsibility of the Expanded Programme on Immunisation, and that eradication could best be achieved in a short time frame through targeted campaigns.^8^

In addition, our respondents commented, central GPEI leadership, particularly at WHO, felt that achieving eradication would demand more accountability, standardisation and quick action than working through government RI programmes could afford. With the aim of achieving the eradication goal quickly, polio campaigns were delivered in parallel to government systems if those government systems were weak.^8 32^

In part, these parallel structures were favoured because they gave global actors more control over on-the-ground activities. Some of our respondents were critical of this orientation. One commented:

If you look at any of [the GPEI’s] actions, the fundamental prism through which they see everything is control. They want to control… They’re very concerned about losing that control, because they want to ensure that polio is eradicated.

Other respondents felt that this way of doing things was integral to polio eradication’s successes:

Could we have designed it differently? Intellectually, we could have. In practical terms and pragmatically, what is unique in polio eradication is the drive, the command and control type of approach and the focus and the verticality to some extent. One can criticize this program for its verticality, on the other hand, it’s been extraordinary, the achievements that have been made.

What our respondents did agree on was that polio eradication was unusually centrally managed, even for a single-disease vertical programme, and that this orientation grew from the eradication agenda.

### From legacy to transition

In the mid-2010s, when legacy planning began, there was broad buy-in to this goal within the programme. Planning for polio’s legacy, however, was complex. Our respondents said that disagreement arose nearly immediately over the word ‘legacy’ itself, reflective of deeper disagreements about what legacy planning should be.

Many of our respondents within GPEI partner agencies such as CDC, UNICEF and WHO felt strongly that morally, the international community should continue to pay for polio infrastructure. But, they said, many within donor agencies felt differently. Some donors feared that the ‘legacy’ terminology would imply they were committing to indefinite funding—something which they did not plan to do. Therefore, many donor agency representatives favoured language clarifying that they were hoping to ‘transition’ the funding of polio infrastructure to someone else. One respondent commented:

We were not ready as polio eradication to finance, and our donors were not ready to finance, staff in polio-free countries that were no longer doing polio. But, these staff needed to be sustained for immunization services, for information systems, for a range of activities that continue to be essential.

The word ‘legacy’ was, therefore, phased out in favour of the word ‘transition’. But many of our respondents commented that the tension between these ideas of what the transition effort was—that it was about sustaining things, but also about ceasing to pay for them at the international level—would dog the transition effort.

In addition to concerns over expectations of sustained funding on the part of donors, some within GPEI were concerned about loss of control within an ongoing eradication effort. Even as the transition effort began, our respondents said that there were many within GPEI who felt that available resources should be focused on eradication and not transition. At that point, in the mid-2010s, the GPEI was facing numerous difficulties, including entrenched wild polio virus transmission in Nigeria, Afghanistan and Pakistan. One respondent commented, ‘It was difficult for [transition] to be on equal footing in the eradication context and push.’ Another respondent reflected:

People felt it was really premature… Like, "We're not done yet. Where is this coming from? We still have the virus raging across the endemics." The outbreaks were just picking up at that point.

Another added:

There were tensions within WHO… because the minute we actually started to [talk about transition], people working on [routine] immunization or transition would say, "Now, *I* have control over this money." And polio people would say, "You don't have control over my money, my polio money, this is money that I need to eradicate polio and I want to control it." This game was actually pretty painful for many of the actors.

### ‘Who’s accountable?’

Amidst these dynamics, planning for transition proceeded. In 2013, the polio Transition Management Group was created, with the goal of supporting counties in planning for transition, and in finding funding to support that transition.

But a second set of tensions arose, centring around the issue of who was accountable for ensuring that transition took place. One respondent commented:

Transition is a move from one place to another, but we don't know what the endpoint is. That’s the difficulty there.

In 2016, the Transition IMB was also created, a parallel body to the GPEI’s powerful IMB. The creation of the TIMB was an attempt to provide direction and accountability to the transition process, moving it forward. However, this proved hard to achieve; as one respondent asked, ‘Who’s accountable?’ Another added:

The GPEI is accountable to the IMB, where it’s less clear that the GPEI is accountable to the TIMB… Who’s responsible to implement these 19 recommendations?

There were, our respondents commented, controversies over not only who was responsible for implementation of transition activities, but also for funding them. At first, the transition effort was financed by the polio programme. But over time, that changed. One respondent explained:

The polio program then started to move away from it, saying, "We're not going to finance transition. We're still financing the staff involved with the polio program and doing a broader range of interventions, but we're not ready to actually finance the actual move of these staff to other programs, we're expecting others to do it."

At the World Health Assembly in 2017, the transition effort was moved out of the GPEI and into a separate WHO initiative. This move, supported by high-level polio WHO leadership, was extraordinarily controversial among our interviewees. Some respondents involved with the decision defended it; one said, ‘I think that it was the right move to make, to actually cut and say, these are volumes of money that need to be identified elsewhere than in the polio programme.’ That respondent continued:

It was more that to actually get a good transition effort going, we needed for it not to be a push by the polio program, but a pull by the other programs… I wanted actually to have the recipient programs such as immunization… understand the risks of the running down of the polio program when polio is eradicated and therefore, prepare for that transition to take over the responsibility, at least, the staff that they felt was needed, the functions that were found to be useful.

Other respondents, however, stated in strong terms that they thought spinning off the transition effort from the polio programme was an abdication of responsibility on the part of GPEI partner agencies. These controversies highlight the difficulties in forming consensus over who was responsible for the transition process itself, and which agencies would take up the mantel.

### ‘How can we collaborate?’

These dynamics at the global level—the command-and-control, eradication-focused orientation of the programme, and tensions over whether polio would own and pay for the transition process—also played out at the national level within countries where the GPEI was active. A major objective of the Transition Management Group was to work with these countries on transition plans. One respondent explained that they ‘identified the 16 countries that were getting 98%, 99% collectively of all of the polio resources, both human and financial.’ Transition plans included a ‘business case’ for transition describing polio ‘assets’ (everything from human resources to lab facilities). These plans would ideally include a budget, details on what programmes would take over these polio assets, and plans for achieving and sustaining the transition.^33^

The extent and detail of this effort reflected a great deal of commitment to the goal by a range of actors at the international level. But our respondents said that the effort proved challenging in practice. One respondent described the experience:

It was just, like, a frantic effort… to count and collect all the assets, as they were calling them, amidst real ongoing eradication…

And then, routine [immunization programs] would get these graphs that were like, "We have 17 million social mobilizers to be used for immunization. Are you excited?" They were like, uhhh…

In many of these countries, the GPEI’s approach over the years had not fostered strong relationships with RI and other key health programme administrators. ‘Polio eradication got so pulled away from the rest of immunisation,’ one respondent explained. Another reflected:

The culture of polio is very different from the culture of other health initiatives. It’s more hands-on, it’s very operational, it’s just doing yourself, whereas the others are much more about building a system, which I think creates—I wouldn't call it tensions, but sometimes it’s as if people just talk across each other. They don't even speak the same language.

One respondent, thinking back, expressed sadness over their inability to reach across programmes to understand the very ‘different ways of thinking about the problem’ in other sectors such as water and sanitation. Another respondent explained:

Programs… weren’t looking at children with the same underserved lens that polio was looking at. *Now*, a lot of people are talking about the underserved and it’s all about the last mile and the last child. But five, ten years ago, that was still really unique…

I think when you were going to people with a social map and that degree of granularity, people were like, “I don't know what to do with this. This is not how we program. We are not at this level of granularity.”

Several respondents commented on the path dependence of transition efforts—they were quite self-critical that they, as polio eradicators, had not built bridges earlier with other programmes. One commented:

Nobody ever said, “Oh, by the way, from a water and sanitation point of view, super interesting piece of information here,” or "From a child protection point of view, you should talk to this religious leader. This is valuable.“… Those early opportunities that might've created demand for transition, I think, there was no space for that, no time for that, no reward for that.

### ‘This ramp down is real’

Like other global programmes, it was a challenge to get governments to finance polio’s infrastructure. Many interviewees commented that the historical verticality of the polio programme meant national governments might not be aware of the potential of polio infrastructure, especially as polio invested in ways that benefited the programme but were not designed to support the health system as a whole.

This could make transitioning even valuable resources difficult. One interviewee reflected:

I think what polio presents as benefits… are not always perceived that way, because polio is perceived as a vertical program. Whereas many others really work through the health systems, through health mechanisms. It’s not about going there and doing things yourself, it’s about really working with the governments to make sure that that happens. It’s a longer-term goal, it’s a longer-term process.

The fact that polio transition marked the end of polio activities, and not the continuation of the programme under country leadership, also marked a difference from other transition efforts. One respondent remarked:

Gavi transition and PEPFAR and others are designed in such a way that, there is a strong system at the end of this that will take things over. GPEI has never done that, because it’s purely on polio. There’s polio, we are there. There’s no polio for the last 10 years, we are out.

However, in part because polio funding had been around for decades in many countries, and in part because international engagement with countries over transition planning was uneven, people at the global level felt many country governments did not really believe polio funding would end that quickly. One respondent commented that they felt they had to deliver a ‘pretty stark’ message to communicate the urgency of the situation. Another respondent commented on the need to convince governments that ‘the ramp down is real’.

Some country governments did see the potential drawdown of polio funds as urgent. One respondent explained:

The South Sudan office really saw the withdrawal of GPEI resources as potentially having such a tremendous impact on the health system there that they wanted to entitle the document “collapse,” what will happen with the withdrawal of GPEI funds.

But not all countries felt the same urgency. Some national governments did not take action until after the money began to dry up. When the funding did end, it could be painful (box 1). One respondent explained:

There were very drastic reductions beginning in 2016, ‘17, ‘18 in the budgets available for the polio free countries…

I think we could be in a much better position if, instead of cutting the budgets of the polio-free countries, go to the donors and say, "We have this tremendous infrastructure and we can use this infrastructure to achieve other major public health successes. Will you help us to do so by providing some sustained funding for these polio-free countries?"… What ended up happening largely in many of the countries, was it was two ships passing in the night.

Box 1 **Ethiopia**

**‘In fact, we built something for polio, but… we're doing much more than polio.’**

The polio programme in Ethiopia is unusual in the extent to which the government successfully used polio funding to shore up other key health system functions over many years. The government used Global Polio Eradication Initiative (GPEI) funding to build out surveillance networks, improve the supply chain and expand human resources for health.^8 41^

This was an effective way to use polio resources to support broader health functions but also left broader programmes vulnerable to rapid funding reductions from the GPEI. Between 2017 and 2020, GPEI funding in Ethiopia decreased by 70%, and the country experienced a 43% staff reduction.^32^ In 2019, government officials said in interviews that they had not been fully prepared to absorb the losses during the GPEI’s 2016–2018 funding drawdown. These reductions led to a rapid loss of surveillance officers, as well as challenges in transitioning functions between personnel, one national-level respondent commented:

A large number of data mangers and officers were reduced from their jobs, so the work loads were transferred to government institutions. But in the government institutions, there is no skilled and experienced human resource, so to manage this all it needs capacity building.

In part, these difficulties were a result of the fact that government salaries were lower than what the GPEI had been paying its staff. Another national-level respondent explained:

The government fee is very low related to the partner’s fee. So, the activities might be stopped. Even if activities don’t stop, the quality of work is under question, or there may be a question of job security.

In the same period as GPEI reductions, the President’s Emergency Fund for AIDS Relief funding for HIV/AIDS control was reduced by 51%.^14^ These rapid, simultaneous transitions put Ethiopia under significant fiscal and material stress.^14^

### ‘Transition to who?’

Countries with relatively robust health systems, like Ethiopia, felt pain when polio funding began to dry up. But in countries with weaker health systems, the effects of the polio drawdown could be even more serious (box 2). In parts of the world with the weakest health infrastructure, the polio programme had to build its most extensive apparatus for campaigns, from cold chain to staff to monitoring systems. In South Sudan and Afghanistan, polio expenditure has constituted over a third of total domestic government expenditure for health.^20^ In some areas, these were the only functioning health services. In South Sudan, one respondent commented, the ‘entire immunisation system’ was largely supported by the GPEI. Thus, there was no system in place to transition polio resources to. One respondent reflected:

There were some rather laughable situations with countries that were in no condition to be able to transition in the way that the development agencies were thinking, namely, like Somalia, South Sudan.

Box 2 **Democratic Republic of the Congo (DRC)**

**‘[The health system] is fragile. It is weak.’**

In the 2000s, after decades of war led to a collapsed health infrastructure, substantial progress was made in the DRC in rebuilding its health system, a challenging task in a geographically large country with limited infrastructure. Yet the situation remains tenuous: one respondent noted in 2019 regarding the health system, ‘It is weak, people are not paid and people are unstable.’

In the same years as the health system was being slowly rebuilt, the DRC was one of the 16 priority countries for the GPEI, and polio money flowed into the country. Polio infrastructure and funding were used to support routine immunisation, surveillance and other key public health functions.

Then, in the transition era in 2015–2016, the GPEI pushed a loud and clear message to the DRC that funding would draw down—and it did, very rapidly, with an over 80% reduction in funding by 2020.^32^ This happened after a long history of heavy dependence on polio funding, and without a strong health system to pick up the slack in the absence of that funding. During that time and since, the DRC has faced repeated outbreaks of circulating vaccine-derived polio.

Another added:

Seriously, I think we have to really make a distinction between where there is potential to transition and where there is a cliff that we need to somehow bridge. Those are cases where there is a transition from basically the GPEI to another external entity, because there’s no government to take over things.

In countries without functioning governments, ‘it’s not going to be a classic transition in the way we have discussed,’ another respondent commented.

A similar but distinct set of issues existed in Nigeria, Pakistan and Afghanistan, the last three polio-endemic countries. In these countries, populations that were chronically underserved by their government health systems saw substantial investments from the polio programme—often hundreds of millions of dollars a year—for decades.

That investment was necessary because the world’s last pockets of polio have been concentrated in areas where government health systems were underfunded or nonexistent (box 3). This set up a striking challenge, as the polio programme had created massive, expensive infrastructures serving the very populations that governments had historically not been focused on serving. Even in cases where governments or other programmes recognised the value of polio infrastructure, then, getting governments to fund it could still be a challenge.

Box 3 **Nigeria**

**‘Yes, we may end up winning the battle against polio, but the wars against other diseases are coming, are we going to be ready for them?’**

Nigeria was the last country on the African continent to be declared polio free, in August 2020. Because polio had been so hard to eradicate, most remained focused squarely on achieving polio-free status, with limited attention paid to transition. In fact, there was resistance to focusing on transition from many who were concerned about ‘finishing the job’.

Because the health system was quite weak in some regions, the Global Polio Eradication Initiative (GPEI) created parallel health system structures in parts of Northern Nigeria. Over decades, these GPEI structures and local health systems became increasingly intertwined, with other programmes piggybacking on the polio platform to deliver services. Also, when community acceptance of polio vaccine became an issue, the GPEI codelivered other health services alongside polio vaccine in campaigns, in an effort to increase acceptance of polio vaccine; these efforts were largely successful.^41^

Our respondents said that the massive GPEI infrastructure in Nigeria translated to a significant business case for transition and reported significant government interest in integrating polio assets into the broader health system. Nigerian government officials envisioned polio transition within the context of developing primary healthcare.^32^

But the challenges were real. In 2019, a number of Nigerian respondents commented that transition planning was made particularly fraught by the fact that the GPEI was paying its staff so much better than the government system. ‘Who is going to be paying these people… working with WHO and given the salary of a senator?’ one respondent asked.

Beyond salaries, the external funding for the GPEI in Nigeria ran broad and deep, from the emergency operations centre to surveillance systems. Our Nigerian respondents were sceptical that the government would be willing to pick up the slack.

Between 2017 and 2020 the GPEI budget was reduced by 81% in Nigeria.^32^ One respondent estimated in 2019 that perhaps 50%–60% of WHO offices were running exclusively on GPEI funding.

Another respondent in Nigeria commented in 2019, ‘You see if this is not handled properly, the entire Nigerian health landscape will collapse, because right now it is the polio program that is keeping the landscape.’ They added sardonically, ‘Me, I’m already making my own transition planning. I’m going into farming.’

### The transition dissolves

These myriad tensions over who would fund continuing activities, and be responsible for planning transition, resulted in the discontinuation of the transition effort. Because of the challenges outlined above, not all priority countries developed transition plans. There was a success story (box 4), but overall the effort had fallen short of the hopes for it.

Box 4 **India**

**‘The project people decided what worked better for them rather than selling out (to external donors). If it is government ownership, then it will work reliably better for the long term.’**

India is the exemplar case of a successful polio transition. In the years leading up to the country’s elimination of polio in 2011, the Government of India began to fund and oversee key polio eradication activities. This government involvement was in part an effort to assert national control over health system activities: as one interviewee explained, ‘government people are fed up with the many donors coming in, and agencies coming in’.

The Indian government’s investments in polio eradication in the 2000s topped US$1 billion; this was unique in countries with extensive polio infrastructures and amounted to more than outlay on routine immunisation (RI) in many of those years.^42^ At that time, India was of critical importance to the global eradication goal, accounting for over 60% of cases globally as of 2009.

Along with this investment came government involvement in, and awareness of the value of, polio structures. In particular, the National Polio Surveillance Project (NPSP), supported through a combination of government and external funding, had what one respondent called a ‘quality stamp’ that led to it taking on broader surveillance activities beyond polio. Increasingly, the government looked to the NPSP to provide broad operational support in developing guidelines and trainings, monitoring health programmes and responding to outbreaks (including, eg, meningitis outbreaks in Uttar Pradesh).

Although the NPSP had external funding and was not initially fully integrated into government structures, the Indian government’s close involvement meant that NPSP staff worked directly with government staff from the beginning, collaborating on tasks from the district to national levels. One respondent who had worked in the NPSP explained:

Now, we hear, and we used to hear at that time also, “These are WHO norms, these are WHO guidelines, this is how WHO works.” In our NPSP, our words were exactly the opposite: “This is what the program needs. This is what is needed for success. These are the gaps we need to plug.” We were very practical and flexible. That was one of the reasons for success.

That is what happened here also in this integration and transition. NPSP, on its own, explored what can be done.

Beyond NPSP, the fact that government officials were involved in polio activities meant that they understood the value of those activities to other programmes—and actively worked towards integration and transition. An exemplar here is Mission Indra Dhanush, an innovative effort to improve RI that one respondent described as ‘leveraging the success of the polio programme’.

Importantly, India’s success in transitioning elements of the polio programme did not occur overnight when polio was eliminated. One respondent explained that, at the beginning, Global Polio Eradication Initiative thought transition plans would be funded and implemented within a couple of years, but as they noted,

That doesn't happen. It didn't happen in India, which was…the best case. We have almost five years of development of a plan and a 10-year plan.

India is an indication that transition can work, but it requires sufficient health system capacity, strong leadership and governance, and a longer time horizon. It also requires real, active internal political will (not simply acquiescence to transition plans) and ongoing external funding: NPSP and other polio assets continue to have substantial funding from WHO.

Critically, too, India’s government had taken over funding and control of the programme in various ways long before the idea of transition was ever discussed, and while polio was still endemic. This successful transition was built on a bedrock of government funding of and involvement in polio activities for many years, supplemented with ongoing external funding.

The ‘premature’ end of the transition effort left many of our respondents angry. ‘They are washing their hands,’ one respondent commented. Another added, ‘I thought that was appalling.’

At its core, several of those people said, the TMG was eliminated because the financial implications of supporting polio resources for the long term were scaring donors. The message, one person involved reported, was: ‘Stop pushing this, because it’s threatening our resource mobilisation.’

‘It was a strange turn of events that we did not anticipate,’ another commented, adding that many within polio eradication wanted to distance themselves from transition because they were fearful that donors would interpret transition efforts as evidence that funding for polio eradication was no longer necessary. This respondent commented on the difference of approach between what they called the ‘eradication zealots’ and those pushing for comprehensive transition efforts:

Was it just the very limited financial transition? Or was it as we, idealist and naïve folks wanted to believe, transition to other priorities where a similar amount of resources could be brought to bear successfully?

In the end, one respondent commented sadly, ‘we ended up closing up shop’.

Yet, while the transition effort was officially over, the issues at play remained: what would happen to polio’s infrastructure once polio was eradicated? Also, as it was clear that circulating vaccine derived polio outbreaks would continue to need outbreak response long after eradication, the GPEI needed to maintain a global presence in some form.

### From transition to integration

Thus, even as transition ended as a goal for the GPEI, another concept appeared to take its place: integration. Many respondents commented that, like ‘legacy’ and ‘transition’, the term ‘integration’ left itself open to differing interpretations. One respondent commented, laughing, ‘Everyone agrees it’s important, but nobody knows what it is.’ Another attempted to provide a workable definition:

Integration meant that we should, to the extent possible, collaborate with other programs to deliver our polio vaccine but ensure that we don't do that in a too-vertical manner, that we associate ourselves with others.

Definitional muddiness aside, what respondents agreed was striking was that integration was agreed on as a goal by a broad coalition within GPEI. This agreement was not total—one respondent said that some within GPEI still saw integration as ‘not our issue’—but it marked a notable shift from the transition era. One respondent commented, ‘I think at least integration right now is at a point that transition has never been at, in that people are finally starting to agree.’

In part this was because it was becoming increasingly clear that in polio’s last strongholds in Afghanistan and Pakistan, it was going to be necessary to provide more services to combat community fatigue. Because integration was potentially critical to the eradication of polio itself, it was a concept with broader appeal. One respondent explained:

Transition is about the future… Integration, to me, is more about how you work. It’s about the present, how the program operates *now*, in a less vertical manner, reaching out to others. It’s more about, how do you get more bang for your buck? How do you actually deliver better services? How do you deal with the fatigue in communities?

This acceptance of integration as a strategy at the highest levels of the GPEI marked a shift. Although support for RI had been a goal on paper since 1988, this had previously been ‘lip service,’ one respondent explained, often without resource investments.

But now the commitment to integration at the highest levels of the polio programme seemed much more real. Our respondents were hopeful about integration—there was wide agreement that it was key not only to eradicating polio but to finding the way forward for polio’s knowledge, human resources and infrastructure once eradication was secured.

‘Let’s re-think and let’s re-speak and let’s re-fund,’ one respondent argued, so that the ‘understanding and commitment we missed’ before—for polio to really support other health services, particularly immunisation—could be achieved.

### Looking back to look forward: lessons for the current transition efforts

Currently, in 2023, the language of ‘transition’ has returned, with a forum held in Geneva to plan for transition, and a draft ‘global vision’ for transition in the works. These draft documents show much learning from the past: there is an emphasis on the need to fund some polio functions, such as surveillance, as global public goods; there is a focus on the need to support national health systems; there is awareness that in some fragile countries, governments cannot take over polio functions; and there is a focus on coordinating donor financing. All of these reflect both a continued commitment to transition, and an effort to avoid repeating the experience of the previous transition era.

In addition, our respondents provided suggestions for what will need to be done now to ensure a transition that sticks.

#### Give up (some) control

A key lesson from the transition era, many respondents asserted, was that the GPEI would have to relinquish some control over its activities, and engage in intentional coordination with other programmes. If polio is to work in partnership with other programmes to achieve integration (and it would have to, because the polio programme does not have expertise in areas like nutrition and WASH), respondents reflected it would be critical to share power over decision-making.

In particular, respondents noted opportunities for working both with Gavi, and with emergency preparedness, as key places where the GPEI could provide real value and build bridges if it was willing to ‘make connections’ across programmes and relinquish a bit of control. To achieve this, respondents argued, would require intentionally moving beyond a way of thinking that assumed a competition for resources.

‘There just needs to be working more hand-in-hand,’ one respondent commented, arguing that eradication of wild poliovirus, stopping circulating vaccine-derived polio outbreaks, and maintaining essential polio functions by surveillance would all be more effective with an integrated approach. ‘But’, this respondent added, ‘they have to rethink command and control’.

The TIMB suggested that although the GPEI might want to maintain its current structure in endemic countries, it could take a back seat in polio free countries.^32^ One interviewee commented positively on this approach, adding that integration should be less GPEI-specific and be managed by the non-polio wings of WHO and other partner agencies. ‘I think the more this is integrated into budgets and not just WHO, I assume it also would have to be UNICEF, the better,’ they noted.

#### Fund integration

The need for funding for integration (both the process and long-term services), our respondents said, was also a key lesson from the previous transition era. There was broad agreement that in many settings, no governments—and potentially no other actors—would step up to fund the polio infrastructure. This was particularly the case in areas where it was most critical, where few other services existed. ‘You have countries’, one respondent commented, ‘that no matter what, will need external support. We need to think through how this external support is delivered, in which form.’

The polio programme itself, most respondents argued, would need to be a major contributor to this funding. Other collaborators will also be needed; one respondent commented, ‘The programme doesn’t have the money to fund the full extent of what integration can and should be.’

Long-term planning at the global level for such funding was a key issue for many respondents. One commented, ‘I would love to see polio completely integrated with the immunisation and emergencies programmes.’ Another suggested:

You can talk about having more and more of the GPEI budget put within the core budget of WHO so that when GPEI is no longer in existence, there’s still that basic budgeting needed for the functions that are important to retain.

Yet funding at the global level was not always easy to translate into action at the local level, particularly if governments did not want to invest in health systems in polio-endemic areas. One official explained that in one polio-endemic country, it was difficult to achieve an integrated approach in practice. While global-level officials tried to push the integrated approach in ‘those districts which are deprived of everything,’ the effort of integration was instead focused in other areas, ‘for political reasons’. As a result, this respondent commented, ‘it hasn’t worked very well.’

Yet despite all these challenges, our respondents saw integration as something of a moral imperative. ‘I think we're at a very critical point where we can make integration happen,’ one respondent commented. In places with ‘limited resources and limited people,’ another respondent argued, ‘polio is shining a light in the darkest corners.’ Respondents wanted the strengths of the polio programme to create broad benefits for some of the world’s most neglected populations.

## Discussion

The GPEI has a high-quality, locally knowledgeable infrastructure in some of the world’s most fragile contexts. Our respondents, across international, national and local levels, were unanimous on the critical importance of a successful transition. Across these various contexts, our respondents saw enormous value in polio structures and argued passionately for the need for continued support and integration of those structures. Yet achieving integration will require close and thoughtful attention to the social and political dynamics at play, with plans to address them effectively.

In particular, four key dynamics require careful attention. The first is the command-and-control orientation of the polio programme, which will almost certainly have to shift if the programme is to integrate successfully. The second is building connections across programmes, both to build relationships and to illustrate the value of polio infrastructure. Third, as this integration proceeds, there is a need for very clear planning to avoid the most painful drawdowns of polio funding.

Finally, in areas where the polio programme has the most extensive infrastructure, it is likely to be infeasible to integrate with other programmes or transition to any other actors. In those areas—in clear need of health services—reimagination may be a more apt metaphor than transition. These areas will require sustained support. The polio programme reaches people that other programmes, governments and health systems neglect. Integrating and providing sustained funding for these services could be a powerful way to increase health equity globally.

These recommendations are challenging. Our interviewees were hopeful that change was possible, although realistic that it would be difficult. It is in this spirit that we offer our recommendations.

This study drew on the experiences and perspectives of people closest to the polio transition effort. Interviewing a broader range of global-level actors might have resulted in somewhat different findings.

There are now many positive steps towards transition, most notably the inclusion of many key polio functions in the general WHO budget for 2022–2023. This funding has been transitioned out of the GPEI for all but 13 countries, a major achievement. This transition of many key GPEI functions to other programmes housed in the Global North does not constitute transition to local ownership, but it is likely a key strategy for keeping some of the GPEI’s most critical infrastructure operational.

Other positive steps include high-level collaboration to fold polio into the broader immunisation agenda; a commitment to co-fund immunisation system strengthening and polio integration in certain countries; and the expansion of the GPEI core partners to include Gavi.^18 19^ There are early positive signs that these efforts may already be strengthening immunisation systems globally.^34^ One example is the significantly strengthened collaboration between the GPEI and RI in Pakistan; of course, this makes continued, long-term investment in transition of even more importance. Another positive effort is the endeavour to collaboratively plan integrated campaigns across a range of vertical programmes, including but going far beyond polio.^35^

Eradication and elimination programmes are innately terminal; some infrastructure should fade out with the end of polio. Yet many functions can and should be integrated. There is evidence of ‘spontaneous’ transition of polio infrastructure, where laboratory and community engagement networks, disease surveillance architecture and human resources have been deployed to fit critical gaps in emergency response, and later retained as part of efforts to build resilient national health systems.^36^ For example, the polio Emergency Operations Centre in Nigeria initially formed the nucleus of the team that promptly responded to the Ebola outbreak in 2014^9^ and has since formed the core of public health emergency management, including playing a major role in responding to COVID-19.

Beyond polio, there are key lessons here for other health programmes as well. Every respondent argued for the critical need for designing for transition in eradication programmes: such programmes should be built from the start to have permanence.

Many respondents argued that the polio programme would have benefited from some transition planning early on, when opportunities to work collaboratively had been bypassed in favour of a vertical approach. In this they echo sustainability and transition scholars who argue that sustainability should be planned early.^1^

The polio transition planning of the 2010s was strong in many of the domains identified as important in the sustainability literature, including financing, service delivery and programming.^37^ Yet it could have benefited from stronger consideration of the social and political elements affecting transition. These include the involvement of local stakeholders in programme design; the integration with existing programmes; and the commitment (or lack thereof) of governments to provide services in areas where the polio programme has the largest footprint.^38^

In their design and implementation, vertical programmes should actively build bridges between disease specific and system strengthening objectives, and set realistic goals for integration. This is easier said than done. It requires working with (and not through) governments and other health system actors; deciding on coordination mechanisms; deciding on what infrastructure will be integrated and how; and tracking progress through robust information systems.^39 40^

What this study shows clearly is that beyond these technocratic steps, it is important to build robust relationships with other programmes, particularly government health systems, to respond to their needs and build collaborations that can eventually form the bedrock of the transition phase of the programme. Programmes built to transition could ensure that their short-term gains can set the stage for more robust health systems down the road.

## Data Availability

Data will be available on reasonable request where confidentiality of respondents can be maintained.
